# Identifying nurses' rewards: a qualitative categorization study in Belgium

**DOI:** 10.1186/1478-4491-4-15

**Published:** 2006-07-06

**Authors:** Sara De Gieter, Rein De Cooman, Roland Pepermans, Ralf Caers, Cindy Du Bois, Marc Jegers

**Affiliations:** 1 Vrije Universiteit Brussel, Department of Work, Organizational and Economic Psychology, Brussels, Belgium; 2 Vrije Universiteit Brussel, Department of Micro-Economics of the Profit & Non-Profit Sectors, Brussels, Belgium

## Abstract

**Background:**

Rewards are important in attracting, motivating and retaining the most qualified employees, and nurses are no exception to this rule. This makes the establishment of an efficient reward system for nurses a true challenge for every hospital manager. A reward does not necessarily have a financial connotation: non-financial rewards may matter too, or may even be more important. Therefore, the present study examines nurses' reward perceptions, in order to identify potential reward options.

**Methods:**

To answer the research question "What do nurses consider a reward and how can these rewards be categorized?", 20 in-depth semi-structured interviews with nurses were conducted and analysed using discourse and content analyses. In addition, the respondents received a list of 34 rewards (derived from the literature) and were asked to indicate the extent to which they perceived each of them to be rewarding.

**Results:**

Discourse analysis revealed three major reward categories: financial, non-financial and psychological, each containing different subcategories. In general, nurses more often mentioned financial rewards spontaneously in the interview, compared to non-financial and psychological rewards. The questionnaire results did not, however, indicate a significant difference in the rewarding potential of these three categories. Both the qualitative and quantitative data revealed that a number of psychological and non-financial rewards were important for nurses in addition to their monthly pay and other remunerations. In particular, appreciation for their work by others, compliments from others, presents from others and contact with patients were highly valued. Moreover, some demographical variables influenced the reward perceptions. Younger and less experienced nurses considered promotion possibilities as more rewarding than the older and more senior ones. The latter valued job security and working for a hospital with a good reputation higher than their younger and more junior colleagues.

**Conclusion:**

When trying to establish an efficient reward system for nurses, hospital managers should not concentrate on the financial reward possibilities alone. They also ought to consider non-financial and psychological rewards (in combination with financial rewards), since nurses value these as well and they may lead to a more personalized reward system.

## Background

Rewards play an important role in organizations: they influence a variety of work-related behaviour [1,2], as well as the motivation of employees [3]. They are used to guide behaviour and performance in an attempt to attract and retain the best-qualified employees and keep them satisfied and motivated [4,5]. As hospitals and other related health-care institutions (e.g. homes for the elderly), are no exception to this rule, rewarding their nurses efficiently and effectively is a challenge for all such organizations, given the crucial influence of nurses on their organizational performance [6-8]. In this study, we concentrate on nurses working in hospitals – the largest group – instead of focusing on nurses working in other health-care institutions. However, we believe our discourse and findings also account for other institutions employing nurses.

Since nurses constitute the largest part of the paid hospital staff, their financial rewards account for a considerable part of the hospitals' budget. Allocating this budget successfully by establishing an acceptable, cost-effective reward system is a true challenge for every hospital manager. However, monetary reward possibilities are much more limited in hospitals than in profit organizations, since the use of financial rewards such as ownership incentives, stock-based-pay and profit-sharing is impossible or inappropriate, due to the non-distribution constraint [9]. Besides, performance-related reward systems are still perceived as difficult to implement in non-profit organizations. If hospitals cannot apply these reward systems, the question is whether they offer or should offer their nurses other non-financial rewards as well.

Earlier studies examining rewards often focused on pay, incentives and benefits, considering money as the only or crucial reward for work [5]. Although many organizations indeed concentrate on financial rewards, other rewards are relevant, too. Special attention to non-monetary rewards is also required [10,11], since the word "reward" does not necessarily imply a financial connotation [5]: Byars and Rue [12] defined rewards as all the returns employees receive as a result of the employment by their organization, monetary as well as non-monetary.

In fact, this discussion on reward typology can be traced back to the original motivation typology of Herzberg [13], identifying intrinsic and extrinsic motivators. Researchers studying rewards adopted this terminology: intrinsic rewards are those generated internally (e.g. personal feelings about the job) and mostly satisfy higher-order needs (e.g. self-actualization). They are derived from factors inherent to the way in which the work is designed, meaning the job content. Extrinsic rewards are externally mediated (e.g. pay) and they essentially satisfy lower-order needs (e.g. safety needs). They are derived from factors associated with the job context [5,14]. Different studies nevertheless acknowledged difficulties with categorizing rewards into these two types [15]. Therefore, this terminology was not applied in the present study.

### Nurses' rewards

In the past, studies identifying nurses' rewards essentially focused on other work-related behaviours or attitudes and included rewards only as an additional variable. They indicated that rewards influence nurses' job satisfaction, their level of professionalism, their performance and their sensitivity to burnout [1,16,17]. A clear focus on the rewards themselves is still rather uncommon in nursing research, possibly illustrating the difficulty of differentiating between motivators and rewards or a belief in the assumption of some researchers that nurses do not let financial payments influence their performance [18,19].

However, Jolner and Hafer [20] concluded that pay seemed to be the most preferred extrinsic reward for nurses. Using a paired-comparison technique, the authors investigated intrinsic rewards derived from the Job Characteristics Model [21]. The extrinsic rewards were formulated by the authors themselves, chosen from the more common reward types used in hospitals: pay, vacation, insurance, days off, hours off and retirement. The nurses in their sample also clearly revealed a preference for the intrinsic rewards of participation and autonomy in the job.

In their study, Hampton & Hampton [1] derived internal and external rewards from earlier studies [22] that had an influence on the level of professionalism and market orientation of nurses as well as on their job satisfaction. Bakker, Killmer, Siegrist and Schaufeli [16] examined the influence of effort-reward imbalance on burnout, by studying the economic reward (in)adequacy of salary and other esteem rewards (appreciation and support from superior and colleagues). These studies clearly identified certain nurses' rewards. However, they all departed from questionnaires presenting rewards to nurses as derived from the general reward literature, without looking at the reward perceptions of the nurses themselves.

Yet recently, nurses' perceptions of economic rewards and their possible impacts have been examined by Kingma [23], using individual interviews, focus groups and observations. She identified two main categories of economic rewards: the financial incentives (e.g. salary and petrol allowance) involved a monetary transfer to the nurses, whereas the financed rewards (e.g. subsidized continuing education and paid sabbatical leave) represented a cost for the employer without any monetary transfer to the nurses themselves. Although nurses did not mention their financial rewards spontaneously, the study results indicated their relevance in the rewarding process.

To conclude, earlier studies identifying nurses' rewards all seem to suffer from one of two restrictions. On the one hand, most studies identifying these rewards essentially examined other core variables, treating rewards as an additional concept. In most cases, they derived rewards from the general reward literature and examined these by means of a questionnaire, with no possibility for particular nurses' specifications. On the other hand, some studies examined nurses' rewards qualitatively, but adopted a narrow interpretation of rewards, concentrating solely on the financial rewards.

With these two limitations in mind, we are not aware of a study specifically concentrating on nurses' rewards and implementing monetary as well as non-monetary rewards. Therefore, the present study will try to identify and categorize rewards that nurses perceive as received for doing their job, starting from the nurses' viewpoint in an attempt to capture as many reward elements as can be used in hospitals and other health-care organizations employing nurses.

## Method

The general research question this study sought to answer was: "What do nurses consider a reward and how can these rewards be categorized?" Since we wanted to start from the viewpoint of the nurses themselves in trying to identify rewards and reward categories, qualitative research was preferred above quantitative techniques [24,25].

### Sample

In-depth semi-structured interviews were conducted with 20 Dutch-speaking nurses working in five Belgian private, non-profit hospitals. Six male and 14 female nurses were interviewed, with a median age of 33 years and a median nursing experience of 11 years. The participants represented all nursing departments (e.g. intensive care, pre-surgery, post-surgery and psychiatric units).

All Dutch-speaking nurses working for a private, non-profit hospital were considered as possible participants. The sampling of respondents started from five acquainted nurses. After 15 interviews, the largest part of the gathered information seemed to be a repetition of the earlier interviews, reaching the information saturation point, so the decision was made to conclude after 20 interviews.

### Interview and questionnaire

The first author contacted the nurses by telephone and briefly explained the context and purpose of the study, before asking them to participate. When a nurse agreed to participate, a time and location for the interview were fixed. Some interviews took place at the respondents' home during their leisure time, while others were performed at the hospital premises before or after the work shift.

An interview guide was composed by the first author, based on the relevant literature and research experience and was tested with three outsiders before starting the interviews. In general, the nurses were asked to describe all rewards they perceived as getting from others in return for doing their job. Each interview lasted around half an hour and was recorded on tape. Afterwards, the interview data were transcribed (average length: 4 pages) and coded.

After the interview, the nurses were asked to complete an additional questionnaire with 34 items derived from the reward literature [12,14,15,20,26-28]. Respondents indicated whether they perceived the item as being a reward for their job or not, by scoring the items on a five-point Likert scale ranging from 1 (not at all a reward for my job) to 5 (definitely a reward for my job). No information about the satisfaction with these rewards was gathered.

### Analyses

First, discourse analysis was applied to the interview data, using the software package ATLAS.ti 5.0. This software makes it possible to use an open coding system based on words and sentences. Reward categories were formed by grouping and integrating related codes, generating new theoretical constructs from the interview data themselves [25,29]. This categorization process was done by the first two authors independently, both categorisations were compared and adjusted after easily reaching a consensus.

After identifying the reward categories, the qualitative data were subjected to content analysis, in an attempt to identify the most important rewards. Importance was defined by the number of nurses who spontaneously mentioned it as being a reward for the job.

Finally, the rewarding potential was also supplemented by the results stemming from the questionnaire data. Due to the small number of respondents, non-parametric statistical techniques (Friedman test, Mann-Whitney test) were performed on these data by using the software package SPSS 12.0.

## Results

The first section presents the results of the discourse and content analyses performed on the interview data. In the second section, the statistical results obtained from the questionnaire data are described.

### Interview results

Analysis indicated two main reward categorization possibilities. First, the codes could be grouped based on the reward source: Who initiates the reward? Different categories were: the hospital superior, patients, colleagues, the nurse her(him)self and outsiders (e.g. family, friends, society in general). The second categorization option was based on the type of reward, which was more in line with the reward identification purpose of this study. Three main reward categories were derived: financial, non-financial and psychological rewards, all containing different subcategories.

Table 1 presents an overview of these reward categories and a definition of their subcategories. For each subcategory, the number of nurses who spontaneously reported it as a reward for doing the job is presented between brackets. The first category, "financial rewards", contains all monetary rewards: monthly pay, on the one hand, and all other remunerations on the other hand, e.g. a New Years' bonus or a vacation allowance.

**Table 1:** Reward categories and subcategories

**Financial rewards**	**Non-financial rewards**	**Psychological rewards**
Monthly pay (14/20) *The take-home pay*	Presents (17/20) *Flowers, presents, chocolates*	Recognition (17/20) *Appreciation shown, respect, win the regard of others*
Other remuneration (12/20) *New Year's bonus, vacation allowance, money from patient*	Human relations support (13/20) *Staff journey, New Year's reception*	Patient contact (14/20) *Relationship with patient, ability to help others*
	General services (7/20) *Vacation, health insurance, free meals*	Compliments (14/20) *Receiving praise, congratulations*
	Individualized advantages (4/20) *Attending training, comfortable work schedule*	Social utility of the work (13/20) *Socially meaningful work, good feeling from job*
		Gratitude (11/20) *Words and other expressions of thankfulness*
		Social support (8/20) *Assistance, countenance, shown sympathy*
		Work climate (5/20) *Positive relationship with colleagues, pleasant working conditions*
		Confidence (4/20) *Trust as shown from the board, possibility to work autonomously*

Financial rewards M = 13/20, Non-financial rewards M = 10.25/20 and Psychological rewards M = 10.75/20

The second category "non-financial rewards", contains rewards with an indirect identifiable monetary value, possibly implying a cost for the hospital, although nurses cannot exchange these rewards for the money itself. This category encompasses four subcategories: presents, human relations support (social support activities organized by the hospital for the nurses), general services (hospital services that apply to all nurses in general) and individualized advantages (services that are considered as a reward by some nurses in particular).

Finally, the third reward category, "psychological rewards", contains eight subcategories: recognition, contact with patients, compliments, the social usefulness of the job, gratitude, social support, work climate and confidence from others.

Content analysis identified the nurses' most important rewards. In general, financial rewards (M = 13/20) were more often mentioned spontaneously than psychological (M = 10.75/20) and non-financial rewards (M = 10.25/20). One nurse explained it like this: "When thinking about rewards, you immediately think about financial things. But if you give it some more thought, we receive a lot more than only these financial rewards" (nurse 9).

From all nurses, 14 perceived their monthly pay as a reward for the job: "Personally, when talking about rewards for doing my job, it is very important for me to be able to earn enough money with it" (nurse 20). In combination with their monthly pay, 12 nurses also seemed to value their other remunerations. Most nurses in our sample perceived their monthly pay as a reward for their job, but it is important to note that the reward potential of money was mainly attributed to its being necessary in order to have a comfortable life: "Your salary is of course a very important reward; without it you can't do anything at all" (nurse 7).

Some subcategories of non-financial rewards seemed to be more important than others. Almost all nurses (17/20) especially valued the presents they receive from others: "Sometimes, even when the evolution of a patients' condition was not that positive at all, we afterwards received a postcard with greetings or a box of chocolates" (nurse 3). Thirteen nurses also valued the reward of human relations support: "Every year, the hospital organizes a New Year's reception for all employees. It is becoming a tradition to thank everybody this way for cooperating successfully and I consider it as a very positive signal" (nurse 14). The other non-financial subcategories "general services" (e.g. health insurance, free meals) and "individualized advantages" (e.g. the possibility of obtaining training, having a good work schedule) were considered as a reward for the job only by smaller numbers of nurses.

With regard to psychological rewards, "recognition" was perceived as a reward by 17 nurses, as important as the non-financial "presents": "For me, the recognition from others is very important. The fact that they like you and that your work means something to them, is a sufficient feeling for me" (nurse 17). Contact with patients was also considered as a reward by 14 nurses, together with compliments from others. Having good contacts with the patients seemed to be of crucial importance: "For me it is a privilege, a reward, that I can be part of the last precious moments of one's life" (nurse 9). Thirteen participating nurses also perceived the social utility of their job as a personal reward, indicating that they like to perform a job that is relevant to people in need and to society in general: "It gives me a good feeling about myself when I can help a patient, help people" (nurse 10). Finally, a smaller number of nurses also perceived gratitude, social support, a good work climate and confidence from others as non-tangible psychological rewards of their job.

In sum, nurses spontaneously mentioned financial rewards more often. However, when looking beyond the reward categories, at subcategory level, the most important rewards indicated were presents and recognition from others, followed by the monthly pay, the contact with patients and the compliments from others and also the human relation support activities as well as the social utility of the job.

### Questionnaire results

The mean scores of the nurses on the 34 reward items are presented in Table 2. They can range from 1 to 5. The reward "appreciation of the work by others" received the highest mean score of 4.20, whereas "participate in recreational activities" received the lowest mean score of 2.60.

**Table 2:** Mean scores on questionnaire items

**Financial rewards**	**M**	**Psychological rewards**	**M**
Pay	3.85	Appreciation of work by others	4.20
Vacation allowance	3.75	Compliment from patient	4.10
Allowance for travel expenses	3.20	Compliment from colleague	4.05
		Respect from colleague	4.00
		
**Non-financial rewards**	**M**	Compliment from superior	4.00
		
Follow training	3.85	Respect from superior	4.00
Good work schedule	3.74	Achieve own goals	3.90
Promotion	3.45	Responsibility	3.85
Job security	3.40	Good contacts with colleagues	3.85
Vacation	3.35	Respect from patient	3.80
Participate to recreational activities	2.60	Pleasant work environment	3.75
		Self fulfilment	3.70
		Involvement in hospital	3.70
		Challenging work	3.70
		Involvement in decision making	3.65
		Help with personal problems	3.65
		Varied work	3.55
		Social standing of job	3.50
		Opportunity to be creative	3.45
		Freedom to make own choices	3.45
		Pleasant working conditions	3.45
		Social utility of the work	3.42
		Personal contacts with patient	3.40
		Autonomy	3.30
		Good reputation of hospital	3.30

First of all, the first two authors independently grouped the 34 items according to the three reward categories identified in the interviews, easily reaching a consensus for each item. A non-parametric Friedman test was performed (Chi^2 ^= 1.33, p > .05), to examine whether these three categories received significantly different mean scores. The rewarding potential of the three reward categories was not statistically different, the average values being 3.71, 3.60 and 3.40, respectively, for the psychological, financial and non-financial rewards.

When looking at the item level, the psychological rewards "appreciation for the work by others", "compliments from patients", "compliments from colleagues", "compliments from superior" and "respect from colleagues", "respect from superior" received the highest mean scores from the nurses. Other important rewards were the financial rewards "pay" and "vacation allowance" and the non-financial rewards "training" and "having a good work schedule".

To examine whether demographical variables raised differences in rewarding potential between groups of nurses (i.e. gender, age, experience), the questionnaire data were subjected to non-parametric Mann-Whitney tests. Concerning age, two independent groups of nurses were formed, based on whether they were above or below the median age of 33 years. For experience, the same procedure was used, based on whether the nurses were above or below the median years of experience of 11 years. Moreover, these groups based on age and experience overlapped perfectly.

Gender did not seem to have any effect on the reward importance, as no significant difference in reward perception was found between male and female nurses. Older and more experienced nurses reported feeling significantly more rewarded by having job security (U = 18.50, p < .05) and working for a hospital with a good reputation (U = 24.00, p < .05) than their younger and less-experienced colleagues. These younger and less-experienced nurses, on the other hand, valued the reward promotion more than the older and more experienced ones (U = 18.50, p < .05).

## Discussion

This study sought to answer the research question: "What do nurses consider a reward and how can these rewards be categorized?". To this end, both qualitative and additional quantitative data were gathered and analysed. Based on discourse analysis, three main reward categories were derived: financial, non-financial and psychological rewards. This categorization is supported by other studies, indicating that non-profit organizations such as the hospitals included in this study offer their employees monetary as well as non-monetary rewards for doing their job [30,31] and that they need both kinds of rewards to motivate their employees [32]. Each of our reward categories contained different subcategories. The importance of these subcategories was examined using content analysis.

If hospital managers want to attract, motivate and retain the best-qualified nurses in an attempt to optimize their hospitals' performance, the need to establish an efficient reward strategy emerges. This study reveals that not only financial rewards matter: nurses also seem to value psychological and non-financial rewards as well. Earlier nursing studies often used general rewards in their questionnaires to examine the influence of these rewards on nurses' performance, burnout and other behaviours, without specifying these rewards to their particular target group [1,16,17,20]. And when they did concentrate on nurses' rewards in particular, they often focused only on financial rewards [23]. The present study, however, looked into the rewarding potential of different aspects of the nursing profession in detail, in order to identify nurses' rewards, which will be used in future studies concerning nurses' performance, commitment and intentions to leave the hospital.

When looking at the reward subcategories defined by this study, the question is whether these reward types are unique for nurses. Indeed, the largest part of the reward types is broadly defined, so these types can also apply to other professions as well. An interesting question for future research may therefore be whether some reward types are more important to particular types of employees than others. We hypothesize that personal contact and the social utility of a job will particularly be perceived as more rewarding by people employed in the non-profit sector.

Balancing between the three reward categories and their subcategories should lead to an optimal reward solution. However, personal preferences should be taken into consideration. Younger and less-experienced nurses, for example, seemed to attribute a higher value to promotion possibilities than their older and more experienced colleagues. These latter nurses preferred a feeling of job security and working for a hospital with a good reputation, instead.

In their study, Jolner and Hafner [20] also suggested an influence of demographic variables on reward preferences: age, seniority and number of children. The older and more experienced nurses in their sample had a higher preference for the retirement reward and additional health insurance than the younger ones. These younger and less-experienced nurses revealed an obvious preference for more days off. Support for this influence of age and seniority on rewarding potential is provided by the Life Structure Theory [33]. Employees in their early adulthood are indeed assumed to value future career successes and status, whereas late adulthood is often a time for reflection, combined with a desire for financial security after retirement.

To conclude, two critical remarks on our work need to be made. The small sample size, due to the qualitative research technique, requires some reservations when generalizing the results. In future research, the reward categorization will therefore be part of a large-scale quantitative validation study. However, no additional rewards were identified after 15 interviews, which made us decide to end the interview series after 20 interviews.

The second remark regards the importance of the non-financial subcategory of "presents". When looking at the interview data, this seemed to be the overall number one reward for nurses. However, this most probably is due to the method used. The interviewing and coding techniques did not permit the precise nature of these presents to be identified: are these presents considered as an expression of gratitude or recognition, or do they have no underlying psychological meaning but only a material value? Therefore, the subcategory "presents" is probably overvalued in this study, whereas some psychological subcategories, already considered as being important rewards, may actually be even more important than revealed here.

## Conclusion

Nurses are one of the most important, if not the most important, human resources of hospitals and other health-care organizations. Their influence on organizational performance is abundantly clear [6,7]. Trying to fulfil their expectations and maximize their performance is a challenging task of hospital managers. Rewarding them sufficiently is part of this deal, since rewards play an important role in attracting, motivating and retaining employees [4,5].

This study identified and categorized all rewards nurses receive for doing their job, starting from the perceptions of the nurses themselves, instead of from the formalized organizational reward systems or from the general reward literature. Indeed, the results indicated that nurses value not only financial rewards, but also non-financial and psychological rewards. Furthermore, rewarding potential and reward preferences also seemed to differ according to age and seniority.

When establishing the most appropriate and cost-effective reward strategy, managers should therefore not rely only on their limited number of formalized financial reward possibilities, but should also acknowledge the value of non-financial and psychological rewards, which can easily be more individualized and thus more effective in stimulating nurses to perform to the best of their abilities.

## Competing interests

The author(s) declare that they have no competing interests.

## Authors' contributions

SDG: design of the study, initiation of the research, gathering and analysing the data, writing the article; RDC: design of the study, co-analysis of the data, editorial revision of earlier drafts; RP: main supervision of the research project, design of the study, editorial revision of earlier drafts; RC: design of the study, editorial revision of earlier drafts; CDB: design of the study, editorial revision of earlier drafts; MJ: supervision of the research project, design of the study, editorial revision of earlier drafts.
